# The Influence of the Unit Junction on the Performance of a Repetitive Structure Micromixer

**DOI:** 10.3390/mi13030384

**Published:** 2022-02-27

**Authors:** He Zhang, Shuang Yang, Rongyan Chuai, Xin Li, Xinyu Mu

**Affiliations:** School of Information Science and Engineering, Shenyang University of Technology, Shenyang 110870, China; yangshuang@smail.sut.edu.cn (S.Y.); chuairongyan@sut.edu.cn (R.C.); lixin@sut.edu.cn (X.L.); muxinyu@smail.sut.edu.cn (X.M.)

**Keywords:** micromixer, multi section repeating structure, unit junction, cube mixing unit, mixing efficiency

## Abstract

In order to investigate the influence of the unit junction on the micromixer performance, a repetitive structure micromixer with a total length of 12.3 mm was proposed. This micromixer consists of a T-shape inlet channel and six cubic mixing units, as well as junctions between them. Numerical simulations show that, when the junctions are all located at the geometric center of the cubic mixing unit, the outlet mixing index is 72.12%. At the same flow velocity, the best mixing index achieved 97.15% and was increased by 34.68% when the junctions were located at different corners of the cubic mixing unit. The improvement in the mixing index illustrated that the non-equilibrium vortexes generated by changing the junction location to utilize the restricted diffusion by the mixing unit’s side wall could promote mixing. Visual tests of the micromixer chip prepared by 3D printing were consistent with the simulation results, also indicating that the junction location had a significant influence on the mixer’s performance. This article provides a new idea for optimizing the structural design and improving the performance of micromixers.

## 1. Introduction

Passive microfluidic mixers have already become an important choice for the pretreatment of samples in micro-total analysis systems (µTAS). T-shaped micromixers with two inlets at 180° or Y-shaped micromixers with any angle (usually less than 180°) are simple structures and are easy to prepare. However, due to the long mixing length and low mixing efficiency, they are rarely used alone, and mostly appear with another micromixer chip as the inlet channel at present. Shakawat et al. presented a tangentially aligned input channels based on the common planar T-shaped inlet that could generate a vertical flow to improve the performance of serpentine micromixer [1]. Zhang et al. [2] designed a T-mixer with swirl-inducing inlets and a T-mixer with rectangular constriction, as well as a T-mixer with swirl-inducing inlets and rectangular constriction to further investigate the inlets’ effects on mixing efficiency, mixing length and pressure drop. Rahbarshahlan et al. [3] investigated mixer inlets with six different geometries and found that the dominant factors in the mixing quality were location, magnitude, rotation direction, the number of vortexes and the Reynolds number. Apart from the factors above, scholars have also paid attention to influencing factors such as the number of inlets [4], the angles of the inlets [5] and the cross-sectional shape of the inlets’ channels [6] on the mixing efficiency. So much research has focused on mixer inlet design, which shows their importance to the mixing effect.

In addition to ingenuity in the design of mixer inlets, scholars have paid more attention to the design of mixing chamber structures. In line with the Cantor fractal [7], Murray’s Law [8] and so on, adding obstacles periodically in the microchannel is a common micromixer design strategy. When flowing through the obstacles, the sample’s streamline produces obvious disturbances, which induce vortices and even turbulence that improve the mixing efficiency. The Dean vortex generated by an arc-shaped microchannel under high Re conditions can also greatly increase the mixing efficiency [9,10,11]. The split and recombine micromixer (SAR) [12,13] first divides the sample into multiple tributaries, then these tributaries are merged together. Repeating the splitting and recombination operation several times can increase the sample’s contact area and thus improve the mixing efficiency. The Tesla mixing structure [14,15] can generate lateral or even reverse tributaries. After reorganization of the lateral tributaries or reverse tributaries, not only does the contact area increase but also the sample radial diffusion reduces and the mixing efficiency is enhanced further. Teardrop and chain mixing structures [16] which use folding, rotation and expansion combined with splitting and recombination also can improve the mixing efficiency.

Chaotic mixing, which was first proposed by Aref in 1984 [17], is one of the most effective passive micromixer design philosophies. This design maintains the basic characteristics of laminar flow, such as low velocity and a small pressure drop, and its chaotic fluid diffusion characteristics are also closer to turbulent flow. Stroock et al. used an interlaced structure at the bottom of the microchannel to successfully induce the initial chaotic flow [18]. Stacking E-shape micromixers and folding E-shape micromixers [19], which combine SAR with chaotic advection mechanisms, can achieve excellent mixing. On the basis of Baker transformation that is isomorphic to Bernoulli transformation, Wang et al. designed a micromixer that successfully induced chaotic flow through repeated compression, stretching, cutting and stacking operations [20]. In the previous work by our group, a micromixer was designed on the basis of plane horseshoe transformation [21] and three-dimensional horseshoe transformation [22], which also successfully induced chaotic flow by multiple “squeeze–stretch” and “bend–fold” operations.

The mixing chamber design strategies described above, with or without added obstacles, Dean Flow, SAR and induction of chaotic flow, have a common feature, namely a periodically repetitive mixing unit structure to improve the mixing results. However, the junction of the two mixing units, which is both the exit of the previous unit and the inlet of the next unit, is not taken seriously in the design process. In this study, the micromixer design starts with the simplest cube mixing units, then a group of micromixers that composed of the same six cubic mixing units and different unit junctions was obtained. To determine the optimal combination, the effect of the unit junction on the mixing effect was studied by numerical simulation. Finally, mixer chips with different junction locations were prepared by using 3D printing technology, and their mixing performance was verified by visual testing technology.

## 2. Mixer Design and Manufacture

**Figure 1:**
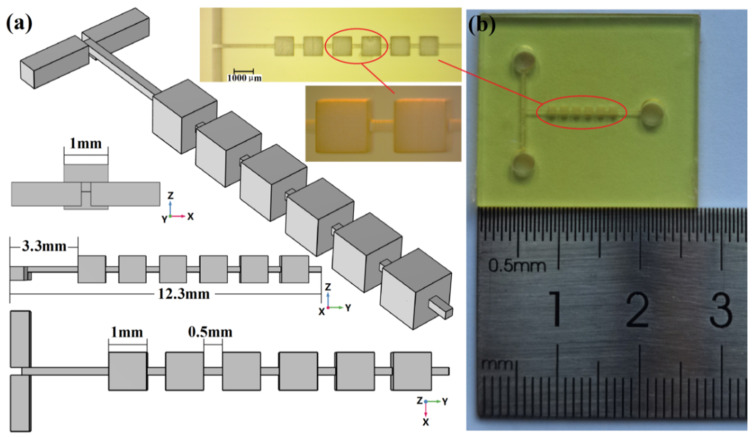
The micromixer with initial repetitive structure. (**a**) The schematics of the mixer; (**b**) photograph of the mixer.

The initial repetitive structure micromixer is shown in Figure 1a.

The mixer consisted of a planar T-shape inlet channel and a mixing chamber that included 6 cubic mixing units as well as junctions between them. The location of the mixer inlet and the outlet as well as the junction between the mixing units were kept consistent. The side length of the cubic mixing unit was 1 mm. The junction channel was 500 μm in length, and the cross-section was square with a side length of 200 μm. In the initial structure, the junction channel was located at the geometric center of the cubic mixing unit. As shown in Figure 1a, the micromixer chip was fabricated by the nanoArch^®^ P150 printing system (BMF Precision Tech Inc., Shenzhen, China). The chip preparation processes were as follows:

1. A series of 2D bitmap files with special patterns were obtained by slicing a 3D structural design drawing of the micromixer.
2. Based on these 2D bitmap files, digital dynamic masks were generated by the digital micro-mirror Device (DMD) in the nanoArch^®^ P150 printing system.
3. When a specific wavelength of ultraviolet light (UV at 405 nm) passed through the digital dynamic masks, the photosensitive resin materials were exposed and cured. One precision structure layer could be produced by one exposure.
4. An integrated micromixer chip was obtained by accumulating the structure after solidification of the layers. Photographs of the micromixer chip are shown in Figure 1b.

## 3. Establishment of the Simulation Model

In order to compare the mixing efficiency of repetitive structure micromixers with different junction locations, numerical simulations are completed by COMSOL Multiphysics 5.6. The basic parameters of the model are shown in Table 1.

According to the model parameters, before the model’s establishment, the Reynolds number (Re) can be calculated by Equation (1). In the mixing unit, the structural characteristic scale (*L*_U_) is 1 × 10^−3^ m, Re = 5 ≪ 2300; in the unit junction, the structural characteristic scale (*L*_J_) is 2 × 10^−4^ m, Re = 1 ≪ 2300.

(1) Re=ρuLη

The calculation of the Re proves that the fluid movement inside the mixer is a typical laminar flow, so the fluid flow of the model can be described by the Navier–Stokes equations as shown in Equation (2). Here, *ρ* is the density (kg/m^3^), *u* is the velocity (m/s), *μ* is the viscosity (N·s/m^2^) and *p* is the pressure (Pa). The modeled fluid is water with a viscosity of 1×10^−^^3^ N·s/m^2^ and a density of 1000 kg/m^3^.

(2) $$\rho u\cdot \nabla u=-\nabla p+\nabla \mu \left(\nabla u+{\left(\nabla u\right)}^{T}\right)\;\nabla u=0$$

The mass transport of the model is described by the convection–diffusion equation (Equation (3)). Here, *D* is the diffusion coefficient (m^2^/s), *c* is the concentration of the components (mol/m^3^) and *R* is the reaction rate between components; *R* = 0 when no reactions occur between the mixing fluids, and the mass transport between fluids is determined by both convection diffusion (u∇c) and molecular diffusion ($D\nabla^{2}c$).

(3) $$D\nabla^{2}c-u\nabla c+R=0$$

After determining the equations, another key step in building the model is to analyze the independence of the mesh. Having the optimal mesh system is important for improving the simulation’s accuracy and saving calculation time. Orderly and clear unstructured tetrahedral units were chosen as the mesh elements. Mixing indexes including the mixer inlet, five mixing unit junctions and the mixer outlet were used to evaluate the mesh’s impact on the simulation’s accuracy. The coordinates of the sampling cross-section along the Y-axis were 3.05 mm, 4.55 mm, 6.05 mm, 7.55 mm, 9.05 mm, 10.55 mm and 12.05 mm. The mixing index was defined as Equation (4).

(4) $$\alpha =1-\sqrt{\frac{\sigma^{2}}{\sigma_{\max}^{2}}}$$

where σ is variance in the concentration, which can be defined by Equation (5); *C_i_* is the concentration of statistical area; *N* = 10 is the number of sample points in the statistical area, with all the sampling points taken from the two diagonals of the sampling cross-section; and $\bar{C}$ = 1.5 mol/L is the average of the statistics.

(5) $$\sigma =\sqrt{\frac{1}{N}\sum_{i=1}^{N}{\left(C_{i}-\bar{C}\right)}^{2}}$$

**Figure 2:**
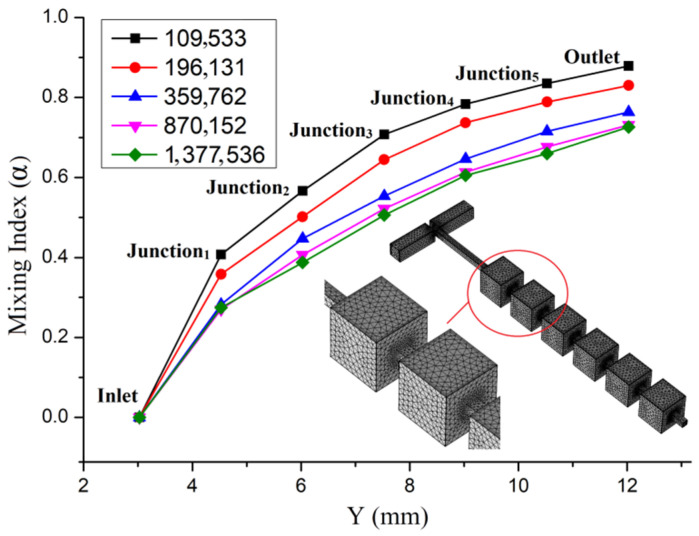
Mesh independence analysis for the mixing indexes along the direction of flow (Y-axis) and the mesh used in the current model.

As shown in Figure 2, when *u* = 5 × 10^−3^ m/s, five mesh solutions with different numbers of nodes ranging from about 109,533 to 1,377,536 were tested for mesh independence. A mesh system with 870,152 nodes was found to be suitable for the current model, because further refinement of the mesh produced less than a 2% change in the mixing index.

## 4. Results and Discussion

### 4.1. Performance Simulation

**Figure 3:**
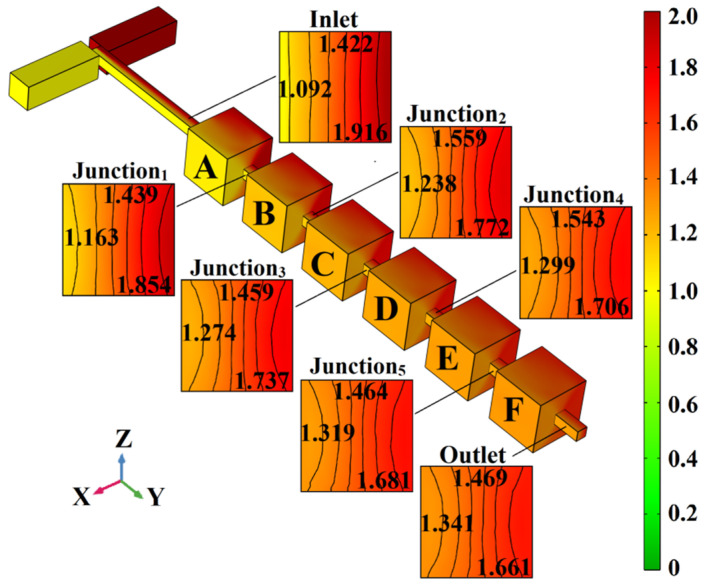
Concentration distributions of the surface and junction cross-section of the micromixer with the initial repetitive structure.

By utilizing the model described above, when *u* = 5×10^−3^ m/s, the concentration distributions of the mixing unit’s surface and the junction cross-section in the initial repetitive structure micromixer were as shown in Figure 3. The mixing fluids are distinguished by color (yellow: 1 mol/L; red: 2 mol/L), while the color gradient between them indicates the degree of mixing.

In Figure 3, the surface concentration of Mixing Unit A can be regarded as an extension of the T-shaped mixing channel, and the cross-sectional concentration distribution of Junction 1 is similar to that of the mixer inlet. At the surface concentration of Mixing Unit B, dark red turns to red, and yellow turns to light orange, but the interface of the two colors is still clearly. The concentration distribution and isoconcentration curve in the junction_2_ cross-section not only clearly show the interface between the two colors, but also a significant difference in concentration (0.534 mol/L). At the surface of Mixing Unit C, the red becomes lighter and the light orange that evolved from yellow becomes orange. The interface between light red and orange is not obvious. However, compared with Junction 2, the cross-sectional concentration distribution of Junction 3 changes a little. At the surface of Mixing Unit D and the cross section of Junction 4, the chromatic aberration is further reduced, and the color interface is not easy to distinguish, but the difference in concentration shown by the isoconcentration curve exceeds 0.4 mol/L. In Mixing Units E and F, although the two colors tend to converge and the interface is indistinguishable, the difference in concentration at the mixer outlet is 0.320 mol/L. Through consideration of the concentration distribution of the mixing unit’s surface and junction cross-section, as well as the mixing index in Figure 2 (*σ*_outlet_ = 72.12%), we can see that the mixing effect of the initial repetitive structure micromixer is not very good.

**Figure 4:**
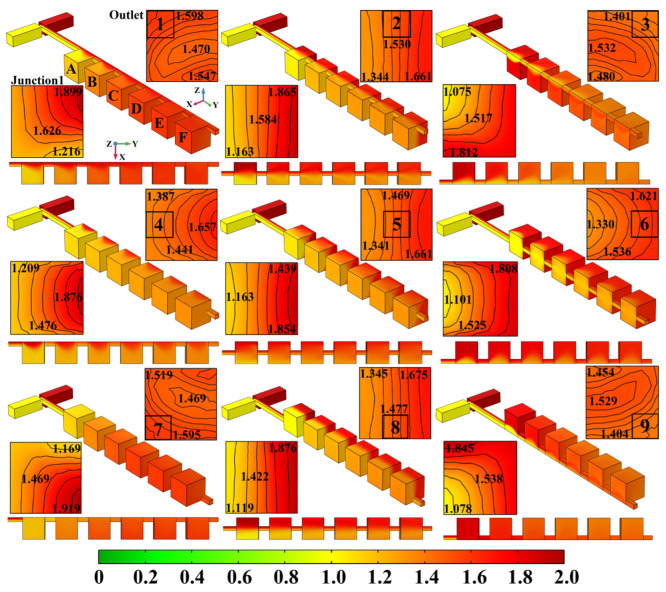
The 3D view, top view and cross-sectional concentration distributions of micromixers with different junction locations.

In order to investigate the influence of the junction’s location on the mixing and improve the efficiency, when *u* = 5×10^−3^ m/s, a series of numerical simulations was carried out on the mixers with different junction locations. The 3D view, top view and the concentration distribution of the surface and outlet of micromixer with different junction locations are shown in Figure 4. The junction locations are given in the cross-section of the mixer outlet. The perspective of the cross-section is from the mixer inlet to the outlet, which is consistent with the flow direction. The nine different locations can be divided into the upper left (Location 1), upper middle (Location 2), upper right (Location 3), middle left (Location 4), true middle (Location 5, the initial structure), middle right (Location 6), lower left (Location 7), lower middle (Location 8) and lower right (Location 9).

From the surface concentration distributions of Location 1, when the fluids enter Mixing Unit A along the positive Y-axis, due to the sudden increase in characteristic dimension of the structure’s cross-section (200 μm turns to 1000 μm), the fluids’ radial velocity reduces (Y-axis), while the lateral (X-axis) and vertical (Z-axis) mass transfer diffusions increase. However, the lateral mass transfer diffusion is mainly along the positive X-axis and the vertical mass transfer diffusion is mainly along the negative Z-axis, since the X-negative and the Z-positive directions are close to the mixing unit’s side wall, where there is not enough space for diffusion. Further, the restricted diffusion is also related to the fluids’ relative location. For example, when the red fluid spreads along the positive X-axis, the mass transfer diffusion occurs between the red fluid and the yellow fluid that is blocking its path. At this time, the lateral diffusion efficiency of the red fluid is mainly determined by the contact area of the two fluids. When the yellow fluid spreads along the positive X-axis, there is no roadblock in its path until it reaches the other side wall of the mixing unit. Along the negative Z-axis, there is no roadblock in the diffusion path until the fluids reach the bottom of the mixing unit, and thus the mass transfer of the two fluids should be the same. However, in the cross-section of Junction 1, there is a significant difference in the vertical mass transfer diffusion along the negative Z-axis. Due to the huge difference in the lateral diffusion, the yellow fluid takes a detour and is the first to reach the bottom of the mixing chamber, where it surrounds the red fluid in the upper left corner of Junction 1′s cross-section. This detour can rapidly increase the contact area of the two fluids. With the repeated mixing units and junctions, the contact area of the yellow and red fluids increases due to the fuller lateral and vertical diffusion, and the mixing of Location 1 is improved. In Unit D, the 3D and top view surface concentration distributions seem consistent, while in the outlet’s cross-section, the encirclement of the red fluid is no longer easy to identify and the difference in concentration reduces to 0.128 mol/L. For Location 2, in terms of lateral mass transfer diffusion, when the red fluid spreads along the negative X-axis in Mixing Unit A, there is no roadblock in its path until it reaches the left side wall. Along the positive X-axis, the mass transfer diffusion is determined by interface area with the yellow fluid. The yellow fluid is the mirror image of the red. Along the positive X-axis, there is no roadblock, while along the negative X-axis, the spread is determined by interface area with the red fluid. For vertical mass transfer diffusion, when spreading along the positive Z-axis, both of the fluids are restricted by the mixing unit’s roof; when spreading along the negative Z-axis, there is no roadblock in the diffusion path until they reach the mixing unit’s bottom. Since the diffusion in the X-direction and the Z-direction is the same, the concentration distributions of the surface and the cross-section of Location 2 are axisymmetric along the Y-axis. Until the end of the six repetition units, the interface of the symmetrical concentration distributions still can be distinguished. The outlet concentration difference of Location 2 is 0.317 mol/L, which is approximately the same as Location 5.

For Location 3, in terms of the surface concentration distributions of Mixing Unit A and the cross-sectional concentration distributions of Junction 1, when the yellow fluid spreads along the positive X-axis, the mass transfer diffusion is restricted by the right-hand side wall, while when it spreads along the negative X-axis, the diffusion is determined by the interface with the red fluid. When the red fluid spreads along the negative X-axis, there is no roadblock in its path until it reaches the left-hand side wall of the mixing unit. When the red fluid spreads along the positive X-axis, the diffusion is determined by the interface with the yellow fluid. When spreading along the positive Z-axis, both of the fluids are restricted by the unit’s roof, but when spreading along the negative Z-axis, the red fluid takes a detour and then surrounds the yellow fluid. Thus, the mass transfer of Location 3 is similar to that of Location 1, but the positions of the yellow and red fluids are exchanged, and the area of encirclement is also changed to the upper right-hand corner of Junction 1′s cross-section. After three mixing units, the interface of the two colored fluids becomes indistinguishable, and the difference in concentration at the mixer outlet reduces to 0.131 mol/L.

For Location 4, in terms of lateral mass transfer diffusion, when the red fluid spreads along the negative X-axis, there is not enough diffusion space because it is too close to the left-hand side wall; when the red fluid spreads along the positive X-axis, the yellow fluid becomes a roadblock again. In terms of vertical mass transfer diffusion, there is no restriction by the unit’s side walls of either the red or yellow fluids along the positive or negative Z-axes. However, due to the difference in lateral diffusion, the yellow fluid still takes a detour and reaches the roof and bottom of the mixing unit first, then surrounds the red fluid. The encirclement position is near the left-hand side wall of the unit, which is different from what was seen in Location 1 and Location3. By the end of six repetition units, the interface of the two fluids near the left-hand side wall can still be distinguished. The difference in concentration at the outlet of Location 4 is 0.270 mol/L.

For Location 5, the initial repetitive structure, in both the X-direction and the Z-direction, the mass transfer diffusion of the yellow and red fluids is consistent. The change in the concentration gradient represented by the colors has been described in detail (Figure 3).

For Location 6, the mass transfer is similar to that of Location 4, but the positions of the yellow and red fluids are reversed and the area of encirclement also changes to the right-hand side wall of the mixing unit. The difference in concentration at the outlet is 0.291 mol/L.

For Location 7, when the red fluid spreads along the negative X-axis, the mass transfer diffusion is restricted by the left-hand side wall, while when it spreads along the positive X-axis, the diffusion is determined by its interface with the yellow fluid. When the yellow fluid spreads along the positive X-axis, there is no roadblock in its path until it reaches the right-hand side wall of the mixing unit. When the yellow fluid spreads along the negative X-axis, the diffusion is determined by its interface with the red fluid. When spreading along the negative Z-axis, both of the fluids are restricted by the unit’s bottom, while when spreading along the positive Z-axis, the yellow fluid takes a detour and then surrounds the red fluid. Thus, the mass transfer of Location 7 is also similar to that of Location 1, except that the area of encirclement is the lower left corner of the mixing unit. The difference in concentration at the outlet is 0.126 mol/L.

For Location 8, when the red fluid spreads along the negative X-axis and the yellow fluid spreads along the positive X-axis, there are no roadblocks in their path until they reach the unit’s side walls. When spreading along the negative Z-axis, both of the fluids are restricted by the mixing unit’s bottom; when spreading along the positive Z-axis, there is no roadblock in the diffusion path until they reach the mixing unit’s roof. Since the diffusion in the X-direction and the Z-direction is the same, the concentration distributions of the surface and the cross-section of Location 8 are also axisymmetric along the Y-axis, and the interface of the symmetrical concentration distribution is visible all through the mixing units. The outlet concentration difference of Location 8 is 0.330 mol/L,

At Location 9, when the yellow fluid spreads along the positive X-axis, the mass transfer diffusion is restricted by the right-hand side wall, but when the yellow fluid spreads along the negative X-axis, the diffusion is determined by its interface with the red fluid. When the red fluid spreads along the negative X-axis, there is no roadblock in its path until it reaches the left-hand side wall of the mixing unit. When the red fluid spreads along the positive X-axis, the diffusion is determined by its interface with the yellow fluid. When spreading along the negative Z-axis, both of the fluids are restricted by the unit’s bottom, but when spreading along the positive Z-axis, the red fluid takes a detour to arrive at the roof first and then surrounds the yellow fluid. Thus, the mass transfer of Location 9 is also similar to that of Location 1, but the area of encirclement becomes the lower right corner of the mixing unit. The difference in concentration at the outlet is 0.125 mol/L.

**Figure 5:**
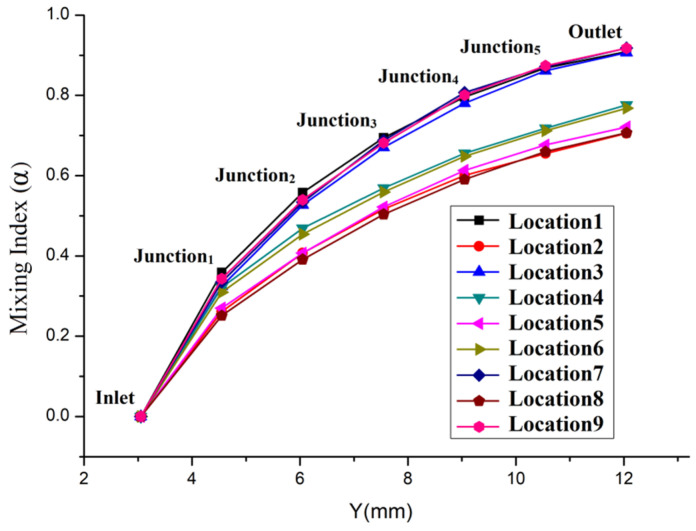
The mixing indexes of the repetitive structure micromixers with different junction locations.

In order to characterize the influence of the junction location on the mixing performance more accurately, according to Equation (4), when *u* = 5×10^−3^ m/s, the mixing indices (α) of the repetitive structure micromixers with different junction locations are given in Figure 5. The location and amount of sampling points are consistent with Figure 2. In Figure 5, the curves can be divided into three groups. Since their curves’ change trends are similar, the mixing indices of Location 1, Location 3, Location 7 and Location 9 are grouped as Group 1. In the curves of Group 1, all the junctions are located at the corner of the mixing unit. The mixing indices of the mixer outlet are α_1_ = 90.89%, α_3_ = 90.66%, α_7_ = 91.78% and α_9_ = 91.76%, respectively, showing that the best mixing results were obtained by placing the junction in the mixing unit’s corner. The mixing indices of Location 4 and Location 6 formed Group 2. In the curves of Group 2, all the junctions are close to one side wall of the mixing unit. The mixing indices of the mixer outlet are α_4_ = 77.62% and α_6_ = 76.83%, respectively. There is a small increase in mixing index compared with the initial location (Location 5, α_5_ = 72.12%). The mixing indices of Location 2, Location 5 and Location 8 make up Group 3. In the curves of Group 3, all the junctions are located along the longitudinal center axis of the mixing unit. The mixing indices of the mixer outlet are α_2_ = 70.56%, α_5_ = 72.12% and α_8_ = 70.66%, respectively, which are the worst among the three groups.

**Figure 6:**
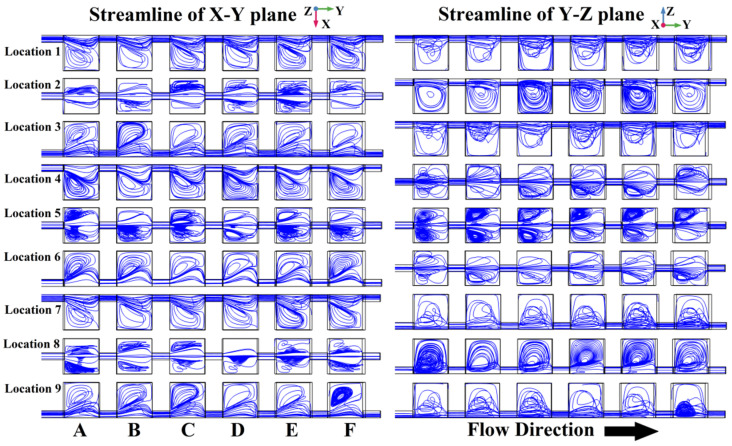
The interior distribution of streamlines in the micromixers with different junction locations.

In the results discussed above, the different junction locations had a significant influence on the repetitive structure micromixer. To reveal the factors of the junction location affecting the laminar flow more clearly, the internal streamlines of the top view (X–Y plane) and the right view (Y–Z plane) are both given in Figure 6. In addition, the magnitude-controlled setting for incompressible flow fields was selected in the simulation software, so that the fluids’ velocity could be reflected by the streamlines’ density.

For Location 1, in Mixing Unit A, in both the X–Y plane and the Y–Z plane, the internal distributions of the streamlines are uneven. Most of the streamlines are concentrated near the pathway formed by the junctions at the upper left corner of the mixing unit. Only some of streamlines appear inside the mixing unit and generate non-equilibrium vortexes in both the X–Y plane and the Y–Z plane due to changes in velocity and direction, respectively, caused by the side walls’ restriction of diffusion. As the mixing unit is repeated, these non-equilibrium vortices also appear repeatedly. Combined with the increased contact area of the two fluids that is also due to repetition of the mixing units, the mixing effect of Location 1 is obviously better. The internal streamline distributions of Location 3, Location 7 and Location 9 are similar to that of Location 1, which also generate non-equilibrium vortexes in both the X–Y plane and the Y–Z plane. The mixing indices of these three structures are also close to that of Location 1, together constituting the Group 1 curves in Figure 5.

For Location 2, in Mixing Unit A, there are vortexes in the Y–Z plane due to a diffusion restriction caused by unit’s roof. These vortices are divided by the junction pathway as the interface (which is also the longitudinal center axis), without an intersection in the X–Y plane. These vortices cannot break the boundary between the yellow and red fluids. With repeated mixing units, the contact area of the yellow and red fluids increases in the Y–Z plane but the boundary is still there. There is almost no increase in the mixing with these equilibrium vortexes, the increase in mixing still mainly relies on the increase in fluid contact area after six repetitions. The internal streamline distributions of Location 5 and Location 8 are similar to those of Location 2, which only generated equilibrium vortexes. Thus the mixing indices of these two structures are also close to that of Location 2, and they constitute the worst performing group (Group 3) in Figure 5.

For Location 4, in Mixing Unit A, there are vortexes in the X–Y plane due to diffusion restrictions caused by unit’s left-hand side wall, but these vortexes do not intersect in the Y–Z plane. These vortexes, which are named quasi-equilibrium vortexes, can break the laminar flow boundary between the yellow and red fluids and thus promote mixing. However the promotion of mixing is limited compared with Location 1, due to the lack of an intersection in the Y–Z plane. As the mixing units are repeated, these quasi-equilibrium vortices also appear repeatedly, and slightly but continuously improve the mixing. The internal streamline distribution of Location 6 is similar to that of Location 4, which can also generate quasi-equilibrium vortexes. The mixing index of Location 6 is also close to that of Location 4, and, together, they constitute the moderately performing group (Group 2) in Figure 5.

Through a comprehensive analysis of the concentration distribution, the mixing index and the streamline distribution of mixers with different junction locations, it can be seen that the restriction of diffusion by the mixing unit’s side wall can force the fluids to take a detour. The detour not only rapidly increases the contact area of two fluids but can also create non-equilibrium vortexes inside the mixing unit. When the junction is placed at the corner of the mixing unit, the fluid detour effect is most significant, and the mixer’s performance is obviously improved.

**Figure 7:**
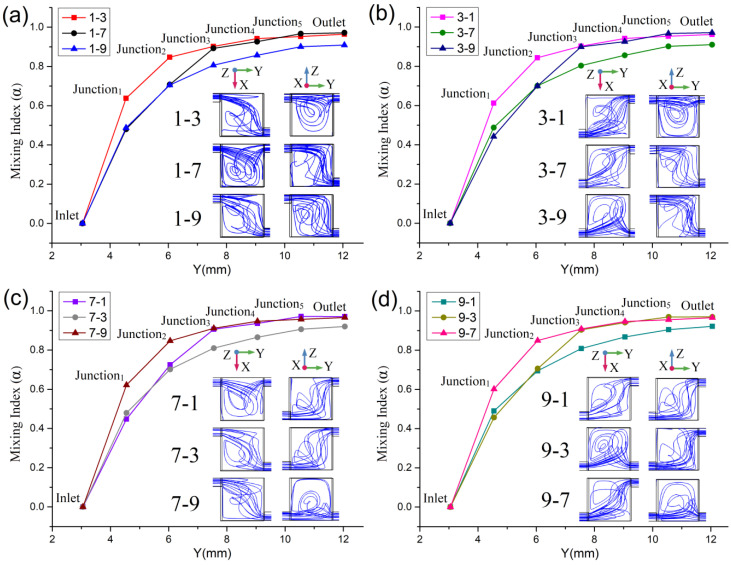
The mixing indexes of repetitive structure micromixers with inconsistent junction location combinations: (**a**) combination of Location 1 and other locations; (**b**) combination of Location 3 and other locations; (**c**) combination of Location 7 and other locations; (**d**) combination of Location 9 and other locations.

This study then investigated whether the repetitive structure micromixer’s performance could be improved by combining inconsistent junction locations. The corner locations (Location 1, Location 3, Location 7 and Location 9) that produced non-equilibrium vortexes were chosen for permutation and combination. According to Equation (4), when *u* = 5×10^−3^ m/s, the mixing indexes (α) of the repetitive structure micromixers with inconsistent junction locations are shown in Figure 7. The location and amount of sampling points are also consistent with those in Figure 2. In order to analyze the influence of the combination of junction locations on the fluid movement, the streamlines inside Mixing Unit A in each case are also given in Figure 7. For the combination of Location 1 and Location 3 (abbreviated as 1–3) in Figure 7a, the mixer inlet, namely the inlet of Mixing Unit A is at Location 1, Junction 1 is at Location 3 because the outlet of Mixing Unit A is at Location 3, while the inlet of Mixing Unit B is also at Location 3. The locations of the inlets and outlets of Mixing Units C–F for Combination 1–3 are identical to those of Mixing Units A and B. Furthermore, Combinations 1–7 and 1–9 in Figure 7a, and Combinations 3–1, 3–7 and 3–9 in Figure 7b, etc. follow the same junction location rules.

In Figure 7a, the best mixing index w obtained by 1–7, for which the mixing index at the outlet α_1–7_ is 97.15%, which is significantly better than that of consistent Location 1 (α_1_ = 90.89%, increasing rate IR_1–7:1_ = 6.89%) or Location 7 (α_7_ = 91.78%, IR_1–7:7_ = 5.85%). The mixing index of Combination 1–3 is α_1–3_ = 96.41%, which is also an obvious improvement. The increasing rates are IR_1–3:1_ = 6.07% and IR_1–3:3_ = 6.34%. However, the mixing index of Combination 1–9 is α_1–9_ = 90.88%, which is slightly reduced compared with the structures with consistent junctions at Location 1 or Location 9. In Figure 7b, the best mixing index is α_3–9_ = 97.13% with the increasing rates IR_3–9:3_ = 7.14% and IR_3–9:9_ = 5.85%. The mixing index of Combination 3–1 is also increased: in this combination, α_3–1_ = 96.21%, IR_3–1:1_ = 5.85% and IR_3–1:3_ = 6.12%. However, in Combination 3–7, α_3–7_ = 91.02%, which means that the mixer’s performance showed almost no improvement compared with Location 3 or Location 7. In Figure 7c,d, the correlations between the junction location and mixing index are similar to those of the two combinations above. When the two corner junctions in the combination are on the same side of the mixing unit, the mixing improves (α_7–1_ = 97.08%, α_7–9_ = 96.58%, α_9–3_ = 96.99%, α_9–7_ = 96.57%). When the two corner junctions are diagonally opposed, the mixing always shows no improvement or even becomes worse (α_7–3_ = 91.99%, α_9__–1_ = 92.12%).

From the streamlines in the X–Y plane view, for example, of Combination 1–3, when the fluids enter Mixing Unit A through the inlet at Location 1, the fluids’ movement along the negative X-axis is restricted. When they spread along the positive X-axis, there is no roadblock in the diffusion path until they reach the other side of the mixing unit. For the combinations with the inlet and the outlet both on the top but not the same side, as well as the injection pressure in the Y-direction, the lateral diffusion fluid seems to move along the diagonal of the mixing unit’s top surface to the outlet. Compared with those for the consistent Location 1 or Location 3, the streamlines in the X–Y plane are elongated, indicating that the flow distance increases and the mass transfer diffusion time also increases. In addition, the streamlines intersect but form no obvious vortexes. According to the streamlines in the Y–Z plane view, when the fluids enter Mixing Unit A through the inlet at Location 1, the fluids’ movement along the positive Z-axis is restricted. When the fluids spread along the negative Z-axis, there is no roadblock in their diffusion path until they reach the mixing unit’s bottom. Therefore, much of the laterally diffused fluids along the diagonal of the top surface also add vertical spreading along the negative Z-axis (these streamlines intersect in the X–Y plane). However, the fluids that diffuse along the negative Z-axis must reverse their direction to flow out of Mixing Unit A through the outlet at the surface. The flow direction reversal along the Z-axis forms non-equilibrium vortexes, and the fluids that participate in the non-equilibrium vortexes are greater than those in the mixer structure with a consistent junction location. Therefore, the main reason why Combination 1–3 can improve the mixing is because the diffusion time and the fluid that participates in the non-equilibrium vortexes both increase.

For Combination 1–7, the mixing enhancement process is similar to that of Combination 1–3. In the X–Y plane, when the fluids enter Mixing Unit A through the inlet at Location 1, the fluids’ movement along the negative X-axis is restricted. When they spread along the positive X-axis, there is no roadblock in their diffusion path until they reach the other side of the mixing unit. However, the fluids that diffuse into Mixing Unit A along the positive X-axis must reverse their direction along the negative X-axis to the outlet near the side wall. The flow direction reversal along the X-axis forms non-equilibrium vortexes. In the Y–Z plane, when the fluids enter Mixing Unit A through the inlet at Location 1, the fluids’ movement along the positive Z-axis is restricted. When the fluids spread along the negative Z-axis, there is no roadblock in their diffusion path until they reach the mixing unit’s bottom. However, driven by the injection pressure, most of the fluid first moves along the unit’s roof to the side wall along the positive Y-axis, and then begin to diffuse vertically along the negative Z-axis. Compared with the diagonal path (the X–Y plane of Combination 1–3), the diffusion distance along the path that consist of two side walls including the positive X and positive Y axes is longer. Similarly, the diffusion time is also longer. The increase in the diffusion path and time allows more fluids to participate in the non-equilibrium vortexes in the X–Y plane of Combination 1–7, and thus more vertically spreading fluids are accompanied by lateral diffusion. Therefore, the mixing index of Combination 1–7 is better than that of Combination 1–3.

For Combination 1–9, when the fluids enter Mixing Unit A through the inlet at Location 1, the fluids’ movement along the negative X and positive Z axes are restricted. When the fluids spreading along the positive X-axis or negative Z-axis, there are no roadblocks in their diffusion path until they arrive at the side wall or bottom of the mixing unit. The mixing unit outlet is at Location 9, which can be reached by spreading along the positive X-axis and diffusion along the negative Z-axis without reversing the direction. Therefore, there are no obvious non-equilibrium vortexes either in the X–Y or the Y–Z plane. Actually, the streamlines of Combination 1–9 stretch along the diagonal of the mixing unit cube, based on the interaction of the injection pressure in the Y-direction as well as the lateral and vertical diffusion. Compared with the consistent junction location near the corner of unit, the diffusion distance and time are both increased slightly. However, there is no improvement in mixing for Combination 1–9, which has no non-equilibrium vortexes. The trends of the mixing index and the change in streamlines shown in Figure 7b–d are similar to those in Figure 7a.

**Figure 8:**
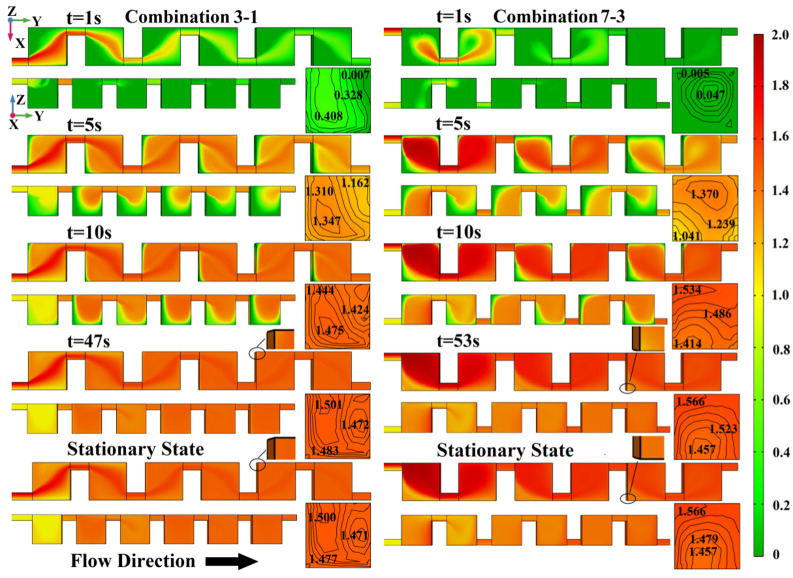
The surface and outlet concentration distributions of mixers with junction locations 3–1 and 7–3 at different time points.

Two different structures were selected to study the dynamic mixing performance: Combination 3–1, which is easy for visualization and experimental observation because all the junctions are located on the upper surface, and Combination 7–3, which is the structure with the junctions located on the cubic mixing unit’s diagonal. When *u* = 5×10^−3^ m/s, the surface concentration distribution of the micromixers with different junction location at different time points are shown in Figure 8. For Combination 3–1, at t = 1 s, the fluids entered all six mixing units quickly through the pathway formed by the junctions near the mixing units’ top surface. However, the surface concentration distribution on the X–Y plane and Y–Z plane is still dominated by green, which indicates the buffer solution with a concentration of 0 mol/L. At t = 5 s, the green in the mixing unit’s top surface is replaced by red and yellow and the colors between them that show the fluid to be mixed. In the Y–Z plane (side view), the green and the other colors are still in a close contest, indicating that the mixing fluid has not completely filled the interior of the mixing unit. At t = 10 s, both the top view and the side view show that the mixing fluids have almost filled the mixing units fully. There is only a small amount of the green buffer solution in the mixing unit’s corners and side edges. At t = 47 s, compared with the stationary state simulation results, there are imperceptible chromatic aberration patches in the mixing unit’s corners. At this moment, the fluid can be approximated as a stationary mixed state. For Combination 7–3, at t = 1 s, the fluids seem to only enter the first four units, as the movement pathway becomes longer, which is caused by movement along the cube’s diagonal. At t = 5 s, the colors that indicate the mixing fluids occupy the dominant position in the mixing unit, but the amount of the green buffer solution is still not small. At t = 10 s, the surface concentration distributions are similar to those of Combination 3–1; there is also a small amount of the green buffer solution in the mixing unit’s corners and side edges. However, the cross-sectional concentration difference of the outlet is 0.120 mol/L, which is a huge increase in performance over Combination 3-1 (0.051 mol/L). At t = 53 s, Combination 7–3 enters a stationary mixing state, but the cross-sectional concentration difference of the outlet is 0.109 mol/L, whereas the stationary mixing state of Combination 3–1 is 0.029 mol/L.

### 4.2. Performance Test

Before the test, the flow velocity *u* = 5 × 10^−3^ m/s was converted to volumetric flow *Q* (m^3^/s) according to Equation (6). Here, *L* is the side length of the equivalent square channel, *η_0_* is the viscosity of the dyed water (8 × 10^−4^ Pa·s at 25 °C) and *ρ* is the density of water (998 kg/m^3^). When *u* = 5 × 10^−3^ m/s, at the micromixer inlet, the structural characteristic scale is 2 × 10^−4^ m, so Re = 1 and *Q* = 9.62 × 10^−3^ mL/min.

(6) Q=ReLη0ρ

**Figure 9:**
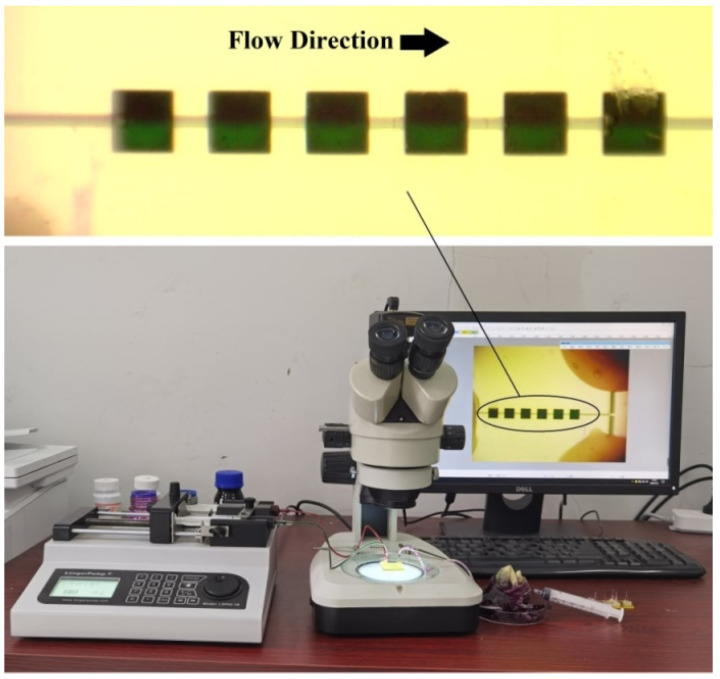
Visual test system and microscopic images of the mixer with junctions at Location 5.

Next, a 1 mol/L Rhodamine B aqueous solution (red) and a 1 mol/L Methyl Green aqueous solution (bluish-green) were utilized as indicators and the viscosity of indicators solution can be approximated as water 1 × 10^3^ N·s/m^2.^ A dual-channel microinjection pump (LSP02-1B, Longer Precision Pump Co., Ltd., Baoding, China) provided the impetus and controlled the volumetric flow. A microscope (XTL-165-VT, Phenix Optics Co., Ltd., Shangrao, Jiangxi, China) was used to observe the mixing progress of the micromixers with different junction locations. The visual test system and microscopic images of the mixer with junctions at Location 5 are shown in Figure 9. In the microscopic image of Location 5, which was used to verify the consistency of the numerical simulation and the visual test, when the fluids had passed through all six mixing units and reached a stationary mixing state, the interface of red and bluish-green was blurred but still distinguishable. The visual test is highly consistent with the numerical simulation, with both indicating that the mixing of Location 5 is not very good.

**Figure 10:**
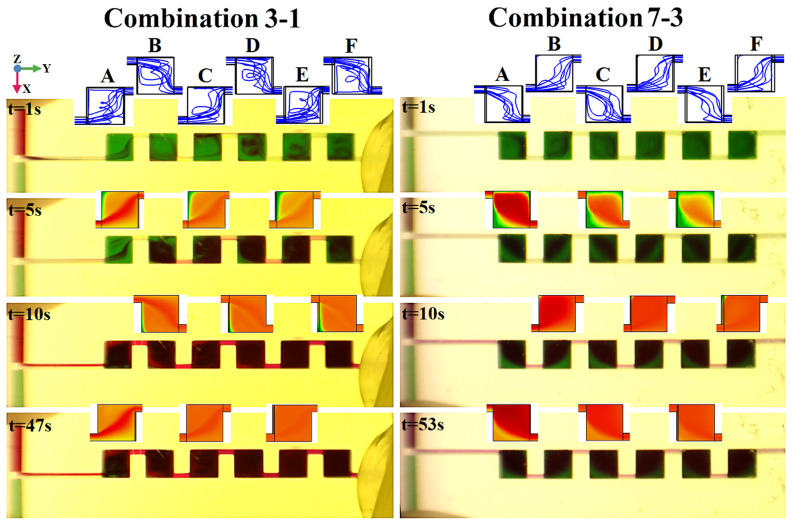
Microscopic images of mixers with different junction location combinations at different times.

With this visual test system, the dynamic mixing test is performed on Combination 3–1 and Combination 7–3. Before the test started, a bluish-green fluid with a higher transparency was used to fill the mixer as the buffer solution. Next, the syringe pump was turned on, and the camera of the microscope recorded the mixing process at different times. Microscopic images of the mixers with different junction location combinations at different times are shown in Figure 10. For a comparison with the simulation results, the streamlines of Combination 3–1 and Combination 7–3 at t = 1 s as well as partial surface concentration distribution of the X–Y plane are also shown in Figure 10.

In the images of Combination 3–1, at t = 1 s, only a small amount of fluid to be mixed enters the mixing unit and the flow trajectory can be observed clearly. The flow trajectory is observed for the red fluid only, since bluish-green fluid was selected as the buffer solution. The red fluid entered all six mixing units, and the flow trajectories were consistent with the simulation streamline, as proved by some salient features, such as the red fluid moving along the diagonal of Mixing Unit A’s top surface to Junction 1, the red fluid moving along the side wall along the positive X-axis in Mixing Unit C, and the vortexes in Mixing Units D, E and F. At t = 5 s, except in Mixing Unit A, we were unable to observe the red fluid’s motion trajectory clearly in the other mixing units, so the surface concentration distributions of the simulation were chosen for verifying the mixing results. There is a certain difference between the surface concentration distributions and the microscopic images because the surface concentration distributions are from the real surface view, while the microscopic images are superpositions of all the concentration distributions in the Z-axis direction. However, there are still many salient features that can prove the consistency of the simulation and experimental results, such as the red concentration distribution belt along the diagonal of Mixing Unit A and the blue-green concentration patches along the negative Y-axis side wall of Mixing Units C and E. In addition, the microscopic image is still dominated by blue-green (the chromatic aberration patches near the mixing units’ side walls are all blue-green), which indicates insufficient mixing at t = 5 s. At t = 10 s, there were still chromatic aberration patches near the side walls of Mixing Units A–D, but these patches had turned reddish. However, there were no obvious chromatic aberration patches in Mixing Units E and F, indicating that the mixing results improved over time. At t = 47 s, the reddish chromatic aberration patches only appeared near the side walls of Mixing Units A and B. There was an imperceptible chromatic aberration between reddish and fuchsia in Mixing Unit C, which is similar to the difference between light red and orange in the simulation’s concentration distribution. The color is a uniform fuchsia in Mixing Units D–F. With a further increase in the mixing time, there is no obvious change in the microscopic image, indicating that the fluids had entered a stationary mixing state.

In the images of Combination 7–3, at t = 1 s, the red fluid entered all six mixing units. However, the trajectories in Mixing Units E and F are not obvious because the red fluids are mainly inside the mixing unit. This is also the reason why there was no surface concentration distribution in the simulation at t = 1 s. However, the consistency of the flow trajectories with the simulated streamline can still be proved by some salient features in Mixing Units A–D, such as the red fluid moving along the side wall along the positive Y-axis to Junction 1 in Mixing Unit A and the vortexes in Mixing Unit C.

At t = 5 s, compared with the concentration distribution of the contemporaneous simulation, there were also many salient features that show the consistency, such as the blue-green patches near the edge, which have been formed by the negative Y-axis and positive X-axis side walls in Mixing Unit A; the blue-green patches near the edge that were formed by the positive Y-axis and negative X-axis side walls in Mixing Unit C. From the other view, the red fluids spread along the diagonal of the mixing unit cube, which is also consistent with the streamline trajectories discussed earlier. At t = 10 s, the blue-green patches near the edge were easily distinguishable, which were formed by the positive Y-axis and positive X-axis side walls in Mixing Unit F. At t = 53 s, the stationary mixing state of Combination 7–3, there were still blue-green patches near the edge that were formed by the positive Y-axis and positive X-axis sidewalls in Mixing Unit F, but these had become imperceptible. With the further increase in the mixing time, there was no obvious further change in the microscopic images of Combination 7–3, indicating that the mixing effect of Combination 7–3 is not as good as that of Combination 3–1.

## 5. Conclusions

In this study, the influence of unit junctions on the performance of a micromixer with a repetitive structure was investigated. A repetitive structure micromixer with a total length of 12.3 mm that consisted of a T-shape inlet channel and six cubic mixing units with junctions between them was selected as the research subject. A series of simulation studies at a flow velocity of *u* = 5 × 10^−^^3^ m/s showed that when the junctions are in the same position and located at the longitudinal axis of the mixing unit, including the initial Location 5, the mixing indices of all three micromixer outlets were around 70%. When the junctions are in the same position and close to the side wall of the mixing unit, the quasi-equilibrium vortexes boosted the promotion of mixing slightly. The mixing indices of both side wall locations were around 77%. When the junctions are in the same position and located in a corner of the cubic mixing unit, the non-equilibrium vortexes generated by the restricted diffusion of the mixing unit’s sidewall promoted mixing. The mixing indices of all four corner locations were greater than 90%, and the best was α_7_ = 91.78%.

By analyzing the performance of mixers with different junction locations, we found that when the junction is placed at the corner of the mixing unit, the mixer’s performance is obviously improved. Next, the four corner junctions were permuted and combined to investigate the effect of inconsistent junction location combinations on the mixer performance. When the two corner junctions in the combinations were on diagonal of the cubic mixing unit, the mixing indices of all four situations had no obvious improvement compared with the consistent corner location. The mixing indexes of Combination 9–1 (α_9__–__1_ = 92.12%) was the best and was slightly better than that of the consistent Location 1 (α_1_ = 90.89%, IR_9__–__1:1_ = 1.35%) or Location 9 (α_9_ = 91.76%, IR_9__–__1:9_ = 0.39%). When the two corner junctions in the combination are on the same side of mixing unit, the mixing index can be improved further because the diffusion time and the fluids that participate in the non-equilibrium vortexes both increase. The mixing indices of all eight same-side corner junction combinations were greater than 96.5%. The best mixing index (α_1__–__7_ = 97.15%), which was significantly better than that of the consistent Location 1 (α_1_ = 90.89%, IR_1__–__7__:__1_ = 6.89%) or Location 7 (α_7_ = 91.78%, IR_1__–__7__:__7_ = 5.85%). Finally, the initial repetitive structure micromixer, the diagonal corner Combination 7–3 micromixer and the same-side corner Combination 3–1 micromixer were prepared by 3D printing technology and then tested by a visual test system. The test results wer consistent with the dynamic simulation, indicating that junction location had a significant influence on the mixer’s performance and the performance of a repetitive structure micromixer can be greatly improved by just changing the junctions’ location.

## Figures and Tables

**Table 1:** The basic parameters of the simulation model.

Parameters Name	Value
Fluid Density (*ρ*)	1 × 10^3^ (kg/m^3^)
Dynamic Viscosity Coefficient (*η*)	1 × 10^−3^ (Pa·s)
Flow Velocity (*u*)	5 × 10^−3^ (m/s)
Viscosity (*μ*)	1 × 10^3^ (N·s/m^2^)
